# Cognitive Sex/Gender Differences and the Beliefs in Own Spatial Abilities

**DOI:** 10.1007/s10508-025-03346-5

**Published:** 2026-02-11

**Authors:** Linda Arrighi, Anna A. Matejko, Markus Hausmann

**Affiliations:** Department of Psychology, Durham University, South Road, Durham, DH1 3LE UK

**Keywords:** Sex/gender differences, Mental rotation, Spatial cognition, Self-perception

## Abstract

Sex/gender differences in specific spatial tasks are well documented, with the male advantage in the Mental Rotations Test (MRT) being one of the largest effects reported in psychological research. Numerous potential contributing factors have been examined, with recent studies highlighting the importance of individuals’ beliefs in their spatial abilities. However, no study has directly manipulated these self-perceptions to assess their causal impact on cognitive performance. The present study investigated whether randomized normative feedback could alter participants’ levels of spatial anxiety, spatial self-efficacy, and spatial self-confidence, and, in turn, affect MRT performance. Participants (*n* = 462; 98 cisgender men, 364 cisgender women) completed two MRT sets and were randomly allocated to receive positive, negative, or no feedback after the first set. Spatial self-perceptions were measured before and after feedback. Results revealed that feedback influenced cognitive performance similarly in cisgender men and women, but cisgender women were generally more likely to lower their spatial self-perceptions, both after feedback and even in its absence. All feedback types affected self-perceptions, but only false positive and true negative feedback affected cognitive performance. Notably, while both types of feedback benefitted cognitive performance, the effect of false positive feedback was mediated by increased self-confidence, whereas the effect of true negative feedback was unrelated to self-perception. These findings suggest that feedback can influence self-perception and cognitive performance through distinct mechanisms depending on its valence and accuracy.

## Introduction

### Sex/Gender Differences in Mental Rotation

Sex/gender differences in cognitive abilities are heavily debated. However, some spatial cognitive tasks have consistently shown significant sex/gender differences (Linn & Petersen, 1985; Reilly & Neumann, 2013; Reynolds et al., 2022; Voyer et al., 1995, 2007, 2017).^1^ One of these sex-/gender-sensitive tasks is the Vandenberg and Kuse Mental Rotations Test (MRT; Peters et al., 1995). The MRT uses three-dimensional cube figures (Shepard & Metzler, 1971) and participants are required to mentally visualize and rotate the figures to determine which two of the four alternatives match a target figure. The MRT has consistently shown the largest cognitive sex/gender difference known in psychological literature (Zell et al., 2015), with men, on average, outperforming women (Voyer et al., 1995). According to meta-analyses (Linn & Petersen, 1985; Voyer et al., 1995), sex/gender differences in the MRT showed large effect sizes (Cohen’s *d*) between 0.56 and 0.73.

Research investigating the origins and underlying mechanisms of sex/gender differences in mental rotation provided empirical support for the effect of biological factors such as sex hormones (e.g., Constantinescu et al., 2018; Hausmann et al., 2000; Hooven et al., 2004; Torres et al., 2006), sociocultural factors such as gender stereotypes (e.g., Guizzo et al., 2019; Halpern & Tan, 2001; Hirnstein et al., 2014; Miller & Halpern, 2014; Moè, 2009; Wraga et al., 2006), as well as psychological variables related to people’s beliefs in their own spatial abilities (herein referred to as “self-perception of spatial abilities” or “spatial self-perception”) such as spatial anxiety, self-efficacy in spatial abilities, and self-confidence in spatial abilities (e.g., Alvarez-Vargas et al., 2020; Arrighi & Hausmann, 2022; Cooke-Simpson & Voyer, 2007; Estes & Felker, 2012; Towle et al., 2005). Biological, psychological, and social factors have been shown to affect cognitive sex/gender differences individually but also via complex interactions between these factors (Hausmann et al., 2009; Rahe et al., 2023). The present study focused on individuals’ self-perception of spatial abilities as potential mediator between sex/gender and spatial cognitive performance.

### Self-Perception of Spatial Abilities

Spatial anxiety, self-efficacy in spatial abilities, and self-confidence are spatial self-perception variables that have previously shown significant sex/gender differences as well as relationships with mental rotation performance. Spatial anxiety was shown to be negatively correlated with mental rotation performance, while both self-efficacy in spatial abilities and self-confidence showed positive correlations (Alvarez-Vargas et al., 2020; Arrighi & Hausmann, 2022; Cooke-Simpson & Voyer, 2007; Estes & Felker, 2012).

Spatial anxiety is a domain-specific psychological trait characterized by negative affect, such as feelings of distress and/or apprehension, when attempting or thinking about tasks involving spatial skills (Lawton, 1994; Lyons et al., 2018; Malanchini et al., 2017; Ramirez et al., 2012; Watson et al., 1988). Self-efficacy in spatial abilities is a psychological trait referring to one’s belief in one’s own ability to successfully solve spatial problems (Bandura, 1994; Towle et al., 2005). Spatial anxiety and self-efficacy in spatial abilities are two constructs which we assume to be highly related, however the extent of their overlap has not yet been investigated. One reason for the hypothesized association between these constructs is that both have independently been found to be associated with MRT performance. For instance, studies have shown higher spatial anxiety to be related to lower mental rotation performance in both men and women (Alvarez-Vargas et al., 2020; Arrighi & Hausmann, 2022). Given that men were generally less spatially anxious than women (Alvarez-Vargas et al., 2020; Arrighi & Hausmann, 2022; Delage et al., 2021; Matthews et al., 2024; Towle et al., 2005), it is not surprising that less spatially anxious men tended to obtain higher mental rotation scores than women (Alvarez-Vargas et al., 2020; Arrighi & Hausmann, 2022). Similarly for self-efficacy, previous studies have shown that lower self-efficacy in spatial abilities was related to lower mental rotation performance in both men and women (Towle et al., 2005) and mediated the well-known sex/gender differences in mental rotation (Arrighi & Hausmann, 2022).

While both spatial anxiety and self-efficacy in spatial abilities are thought to be (1) relevant for spatial tasks more broadly, (2) relatively stable in adulthood (Muffato et al., 2023) and, plausibly, (3) somewhat overlapping psychological domain-specific traits, self-confidence is conceptually distinct from both. Specifically, self-confidence is a state variable which captures individuals’ momentary evaluation of their own performance at the item level and is therefore state- and task-specific. Self-confidence in the MRT has been shown to predict individuals’ MRT performance, with more self-confident men showing higher MRT performance than women, who were generally lower in self-confidence than men (Arrighi & Hausmann, 2022; Cooke-Simpson & Voyer, 2007; Estes & Felker, 2012). However, this effect has not been replicated in other spatial tasks such as paper folding (a measure of spatial visualization), spatial relations test (a measure of 2D spatial orientation), and the Purdue Spatial Visualization Test (a measure of 3D spatial orientation; Ariel et al., 2018). Despite significant sex/gender differences in self-confidence (i.e., men being, on average, more self-confident than women), men and women did not perform significantly differently in any of the spatial tasks in this study (Ariel et al., 2018). This suggests that, while studies investigating the MRT have frequently shown sex/gender differences in self-confidence as well as in actual cognitive performance (Arrighi & Hausmann, 2022; Cooke-Simpson & Voyer, 2007; Estes & Felker, 2012), sex/gender differences in self-confidence may not necessarily correspond with sex/gender differences in actual cognitive performance in other (spatial) cognitive tasks. A recent study has also shown that sex/gender differences in spatial self-confidence may be related to women underestimating their abilities, as men tended to be accurate in their perception of their own spatial abilities while women underestimated them (Hofer et al., 2025).

Overall, findings from previous studies suggest that sex-/gender-sensitive spatial self-perception variables such as spatial anxiety, self-efficacy, and self-confidence can significantly contribute to sex/gender differences in mental rotation performance (Alvarez-Vargas et al., 2020; Arrighi & Hausmann, 2022; Estes & Felker, 2012; Towle et al., 2005). However, no study has yet assessed their discriminant validity or directly manipulated self-perception of own spatial abilities to observe whether such manipulation would lead to changes in cognitive performance. The current study aimed to replicate the well-documented sex/gender difference in MRT performance (Linn & Petersen, 1985; Voyer et al., 1995; Zell et al., 2015) and directly manipulate levels spatial anxiety, self-efficacy, and self-confidence through performance feedback, and observe corresponding effects on sex/gender differences in MRT performance.

### The Effect of Feedback

Feedback is formally defined as providing information about some aspects of one’s task performance (Kluger & DeNisi, 1996). While the amount of information provided can vary, feedback may contain information about the following: whether answers were correct or incorrect; what the correct answers were; explanations of what makes certain answers correct; how an individual’s performance compares to that of specified others (or normative feedback, which the current study employed; Kluger & DeNisi, 1996). According to previous reports, feedback can significantly affect self-confidence and cognitive performance (Estes & Felker, 2012; Kluger & DeNisi, 1996; Lenney, 1977; Lovász et al., 2022; McCarty, 1986; Peifer et al., 2020; Rahe et al., 2019). A recent meta-analysis showed that feedback interventions, such as providing participants with performance feedback—even if it was false or randomized feedback—positively affected cognitive outcomes (*d* = 0.51), which included student achievement, memory retention, and cognitive test performance (Wisniewski et al., 2020). However, this was a heterogeneous effect substantially affected by the amount of information provided, with more information leading to larger improvements in performance (Wisniewski et al., 2020). Feedback also positively affected motivational outcomes (*d* = 0.33), which included intrinsic motivation, self-efficacy, and persistence (Wisniewski et al., 2020). According to motivation theory, the effect of feedback on cognitive performance can be explained by its effect on intrinsic motivation, self-efficacy, and persistence (Ryan & Deci, 2000). In line with this, a study showed that the improvement in mental arithmetic performance after randomized normative positive feedback was mediated by task-related self-efficacy, in both men and women (Peifer et al., 2020). In contrast, a negative effect on motivational outcomes is expected when feedback is negative and uninformative (Ryan & Deci, 2000). Indeed, a previous meta-analysis showed that negative feedback, when compared with positive feedback, decreased intrinsic motivation (Fong et al., 2019). Overall, it has been hypothesized that the effect of feedback on cognitive performance may be indirect through motivation and self-efficacy, however only a few reports have examined this mediating mechanism directly, and none on mental rotation performance specifically.

In terms of sex/gender differences, a recent study found that feedback in the form of encouragement differently affected men’s and women’s performance in an online visual perception game (Lovász et al., 2022). Specifically, encouragement improved women’s and reduced men’s cognitive performance. In addition, the effect of encouragement on game performance was stronger in participants with lower baseline task-related confidence. This suggests that baseline self-perception may moderate the effect of feedback on cognitive performance for both men and women. Additionally, a study on probabilistic learning (Tobias & Ito, 2021) found that baseline general anxiety led to stronger electrophysiological responses (i.e., increased amplitude of event-related potentials in the brain measured after error commissions and feedback presentation) to negative cues such as errors and negative feedback, with stronger effects in women than men. Another report used randomized normative feedback to manipulate men’s and women’s confidence in their abilities to complete a creativity task (McCarty, 1986). They found that, while men were overall more self-confident than women regardless of feedback condition (positive, negative, no feedback), both positive and negative feedback effectively manipulated men’s and women’s self-confidence in their creative abilities. Numerically, positive feedback improved both men’s and women’s self-confidence and negative feedback reduced men’s but improved women’s self-confidence, however this study did not statistically test whether the effect of feedback on self-confidence was sex-/gender-specific (McCarty, 1986). Overall, while previous studies showed that feedback can affect men’s and women’s cognitive performance and self-confidence differently, it is unclear to what extent the effect of feedback on cognitive performance is direct / indirect through self-perception variables, such as anxiety, self-efficacy, and self-confidence.

### The Effect of Feedback on Mental Rotation

Only very few studies have investigated the effect of feedback in the context of spatial cognitive abilities (Estes & Felker, 2012; Rahe et al., 2019). Estes and Felker investigated the effect of randomized normative feedback on MRT performance in adults and found that both men and women who were told that their performance in a line judgement task was above average (positive feedback) went on to obtain higher scores in the MRT compared to those who were told that their performance was below average (negative feedback). The interaction between feedback condition and sex/gender was not significant, indicating that feedback affected men’s and women’s MRT performance similarly. It is important to note, however, that although significant sex/gender differences in the MRT were found in both feedback conditions (i.e., men outperformed women), the effect size was smaller in the negative feedback condition. Critically, spatial self-perception variables such as self-confidence were not measured in this study. Finally, Rahe et al. (2019) provided adult men and women with accurate feedback (i.e., whether the response was correct or not) after each item in a chronometric mental rotation task. Participants in the control condition performed the same task, but no feedback was provided. This study found that women in the feedback condition were faster to respond to mental rotation items compared to women in the control condition. Feedback did not affect men’s reaction times. In contrast to participants of the feedback condition, who perceived the mental rotation task as similarly difficult regardless of sex/gender, women in the no feedback condition perceived the task as more difficult than men. This finding suggests that providing accurate feedback may motivate women to respond more quickly to mental rotation items, which may lead to women perceiving the task as easier. Critically, spatial self-perception variables such as spatial anxiety, self-efficacy, and self-confidence were not measured in this study.

In summary, only two previous studies have investigated the effect of feedback on mental rotation performance, and the open question of whether the observed effects of feedback were direct or indirect through spatial self-perception variables remains unaddressed. While there is some literature on the topic of feedback effects on math performance and math anxiety (e.g., Balt et al., 2022), we were specifically interested in cognitive tasks that are highly sensitive to sex/gender differences, where performance patterns may be confounded by non-cognitive factors also influenced by sex/gender.

### Rationale and Hypotheses

The current study aimed to investigate the effect of randomized normative feedback (positive, negative, no feedback) on sex/gender differences in MRT performance, and the extent to which this effect is mediated by spatial anxiety, self-efficacy, and self-confidence. Men’s and women’s MRT performance was tested with the Revised Vandenberg and Kuse Mental Rotation Test (version MRT-A), containing two sets of 12 items each (Peters et al., 1995). Participants’ self-perception of spatial abilities was measured with three questionnaires and was directly manipulated by giving participants randomized normative feedback (positive, negative) or no feedback halfway through the MRT.

We hypothesized sex/gender differences in all variables, with cisgender men showing higher MRT performance and better spatial self-perception compared to cisgender women (Hypothesis 1). We also predicted positive feedback to be related to better spatial self-perception (spatial anxiety, self-efficacy, and self-confidence) and higher MRT performance after compared to before feedback (Hypothesis 2), and negative feedback to be related with worse spatial self-perception and lower MRT performance after compared to before feedback (Hypothesis 3). Given that we predicted sex/gender differences in all dependent variables, we further hypothesized the effect of feedback to be particularly pronounced in cisgender women (Hypothesis 4). Finally, it was hypothesized that spatial self-perception variables (spatial anxiety, self-efficacy, and self-confidence) would mediate the effects of positive and negative feedback on MRT performance (Hypothesis 5).

## Method

### Participants and Design

Overall, 664 participants were recruited for this online study: 106 males, 378 females (sex assigned at birth), and 180 participants whose assigned sex was unknown because they withdrew before being presented with demographic questions. Of the total sample, 499 participants were recruited through the Department’s student participation pool, 63 participants via Prolific, and 102 participants by emailing students from other psychology departments across the UK. All participants were eligible to enter a free prize draw to win one of nine £10–20 vouchers, and psychology students from Durham University additionally received course credit for participation. Pre-screening excluded individuals with psychiatric, endocrine, cardiovascular, or other specific chronic conditions. Participants were naïve to the study hypotheses and its sex/gender focus. Written informed consent was obtained from all individual participants, and their privacy rights were upheld.

A total of 202 participants were excluded from all analyses for the following reasons: (1) non-completion of the online material and, critically, failure to provide assigned sex and gender identity information (*n* = 180), (2) poor performance on the MRT (score of zero, *n* = 10), or (3) identification with a gender that did not align with their assigned sex, or failure to provide gender identity information (*n* = 12). The final sample comprised 462 participants (98 cisgender men, 364 cisgender women). Cisgender identity was defined as alignment between gender identity and assigned sex at birth (i.e., man and assigned male at birth, woman and assigned female at birth). Cisgender men were significantly older (M ± SD = 21.5 ± 4.12 years, range: 18–44) than cisgender women (19.9 ± 2.97 years, range: 18–41), *t*(125.41) = 3.49, *p* < .001, *d* = 0.48, however, the mean age difference (1.6 years) was small. Including age as a covariate in the analyses did not yield any main effects or interactions, so ANCOVA results are not reported here. Detailed demographic information is provided in Table 1.

**Table 1:** Demographic information listed as absolute frequencies (and percentages)

Characteristics	Cisgender Men	Cisgender Women
***N***	98	364
**Field of study/career**		
Psychology	74 (75.5)	325 (89.3)
STEM^a^	13 (13.3)	23 (6.3)
Natural and Applied Sciences^b^	6 (6.1)	3 (0.8)
Social Sciences	1 (1)	8 (2.2)
Humanities	3 (3.1)	2 (0.6)
Other^c^/Missing	1 (1)	3 (0.8)
**Education**		
Secondary (GCSE)	4 (4.1)	4 (1.1)
High School (A levels)	68 (69.4)	287 (78.8)
Bachelor’s Degree	25 (25.5)	63 (17.3)
Master’s Degree	1 (1)	10 (2.8)
**Ethnicity**		
White	67 (68.4)	218 (59.9)
Asian	15 (15.4)	104 (28.6)
Black	8 (8.1)	12 (3.3)
Mixed/Other/Missing	8 (8.1)	30 (8.2)
**Sexual orientation**		
Heterosexual	72 (73.5)	267 (73.4)
Gay/Lesbian^d^	10 (10.2)	17 (4.7)
Bisexual/Pansexual^d^	11 (11.2)	52 (14.3)
Asexual^d^	0 (0)	4 (1.1)
Prefer not to say/Other/No answer^d^	5 (5.1)	24 (6.6)
**Previous mental rotation experience**		
Naïve to mental rotation tasks	49 (50)	174 (47.8)
Not naïve to mental rotation tasks	48 (49)	184 (50.5)
No answer	1 (1)	6 (1.7)

^a^ STEM = Science, Technology, Engineering, Mathematics

^b^ includes Biology

^c^ i.e., services; logistics; chef; and management

^d^ Grouped together and referred to as sexual orientation diverse in the Discussion

This study employed a 2 (Gender: cisgender men, cisgender women) × 2 (Time: before feedback [T1], after feedback [T2]) × 3 (Feedback: positive, negative, none) mixed design. A priori power analysis (alpha of 0.01, power of 0.99, medium effect size of *f* = 0.25) indicated a minimum total sample of *n* = 117 for a repeated ANOVA examining within-between interactions (G*Power v3).

### Procedure

**Fig. 1:**
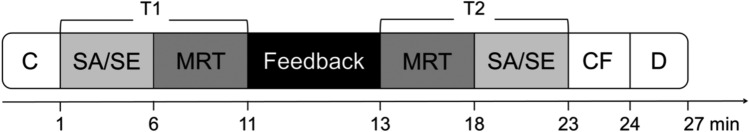
Detailed procedure of study. T1 = Timepoint 1; T2 = Timepoint 2; C = Consent; SA = Spatial anxiety; SE = Self-efficacy in spatial abilities; MRT = Mental Rotations Test (Peters et al., 1995) with self-confidence ratings; CF = Credibility of feedback; and D = Demographic information. Times in minutes are approximate

This study was conducted online via Qualtrics. Before cognitive testing (T1), participants completed two questionnaires in counter-balanced order: one assessing spatial anxiety and the other assessing self-efficacy in spatial abilities. Participants then completed one of two MRT sets, providing self-confidence ratings after each item. Participants were randomly assigned to one of the three feedback conditions: positive, negative, or none. In the positive (and negative) feedback conditions, participants read the following message after the first MRT set: “Please read the information contained in this page carefully. Your performance has been evaluated in terms of accuracy and rapidness. According to this analysis, you have performed better than 90% (worse than 90%) of the previous participants. Compared to the average participant, you will be better (less) able to follow new instructions and to manage these types of problems spontaneously” (adapted from Peifer et al., 2020). The feedback was displayed for 2 min, followed by a 1 min break. Control participants only saw the break page for 1 min. Participants then completed a second MRT set with self-confidence ratings after each item and repeated the spatial anxiety and self-efficacy in spatial abilities questionnaires. Finally, participants rated the credibility of the feedback, completed demographic questions, and were debriefed. The study took approximately 45 min to complete. A detailed overview of the procedure is shown in Fig. 1.

### Measures

#### Spatial Anxiety

Spatial anxiety was assessed using the scale by Arrighi and Hausmann (2022), which measures affective responses to spatial problems. Participants rated 12 statements (presented in counter-balanced order) on a 7-point Likert scale (1 = *do not identify at all*, 7 = *strongly identify*). Seven items were reverse coded prior to computing the mean score (range, 1–7). Cronbach’s α was 0.87 in the normative sample (Arrighi & Hausmann, 2022) and 0.81 in the present sample at T1.

#### Self-Efficacy in Spatial Abilities

Self-efficacy in spatial abilities was measured using a 12-item scale assessing participants’ perceived competence in their spatial ability. Items were presented in counterbalanced order and rated on a 7-point Likert scale (1 = *not at all confident in my ability*, 7 = *highly confident in my ability*). The 12 items included: Item 1. I have a good sense of direction. Item 2. Drawing a map of the area where you live. Item 3. Reading road maps. Item 4. Giving directions (written or oral). Item 5. Locating North (without a compass). Item 6. Drawing a map of the United Kingdom. Item 7. Driving to a destination without the aid of directions. Item 8. Recognizing complicated drawings when viewed upside-down. Item 9. Imagining common objects from different perspectives. Item 10. Imagining common objects and rotating them mentally. Item 11. Imagining abstract objects and rotating them mentally in all directions. Item 12. On this specific mental rotation task. The mean score was used for analysis (range 1–7). Cronbach’s α was 0.93 in the normative sample (Hausmann, 2026) and 0.89 in the present sample at T1.

#### Mental Rotation Test

MRT performance was assessed using the MRT-A version of the Revised Vandenberg and Kuse Mental Rotations Tests (Peters et al., 1995). The test consisted of two 12-item sets, presented in counterbalanced order across participants, with one set administered before (T1) and one after (T2) the feedback manipulation (or no feedback in the control condition). Each item displayed a target figure, located on the left, and four stimulus figures, located on the right. A correct response required identifying both matching stimuli, yielding a maximum of 12 points per set. The MRT was timed, with 4 min per set (8 min in total) to allow for self-confidence ratings, and participants could skip items.

#### Self-Confidence in MRT Performance

Following Estes and Felker (2012), participants rated their confidence in their previous answer being correct after each MRT item on a 7-point Likert scale (from 1 = *not at all confident* to 7 = *extremely confident*). Only ratings from attempted MRT items were included in the analysis.

#### Credibility of Feedback

Feedback credibility was measured on a 7-point Likert scale (1 = *strongly disagree*, 7 = *strongly agree*) in response to “How much do you agree that the feedback you received after the first set was truthful?” Participants who responded with 4 (*I am not sure*) or below were asked about when they first doubted the feedback’s credibility. Response options were: (1) when you received the feedback, (2) when you started the second set, (3) during the second set, (4) after the second set, or (5) when you were asked if you thought the feedback was truthful.

## Results

Statistical analyses were conducted in SPSS v29 for Windows. The raw data files are available on the Open Science Framework (Arrighi et al., 2023).

### Credibility of Feedback

As positive and negative feedback were provided to participants at random and without considerations of prior performance, we first conducted a series of exploratory analyses to evaluate whether participants perceived the feedback as credible and check the relevance of controlling for feedback credibility and/or accuracy in subsequent analyses. The initial sample (*n* = 462) was reduced by excluding participants who did not complete the credibility questions (*n* = 3) and those assigned to the control condition (*n* = 103), resulting in a final sample of 356 participants (*n* = 76 cisgender men, *n* = 280 cisgender women). Mean credibility ratings were 3.45 ± 1.83 on a scale where 4 (*I am not sure*) represented the midpoint, indicating that participants generally regarded the feedback as lacking credibility. This pattern suggested that the feedback manipulation may have been insufficient to elicit strong perception of authenticity or trustworthiness.

#### Feedback Accuracy

Given the generally low credibility ratings, we examined whether this pattern might be attributable to the accuracy of the feedback provided. Feedback accuracy was determined by comparing participants’ baseline (T1) mental rotation performance to the feedback they received. A median split of the full sample (*n* = 462; *Mdn* = 4) classified participants as high performers (T1 MRT score ≥ 4) or low performers (T1 MRT score < 4). Participants were then assigned to one of three feedback accuracy conditions (i.e., true feedback, false feedback, or control) based on the alignment between their performance level and the valence of the feedback (e.g., high performers receiving positive feedback were categorized as receiving true feedback).

When the control condition was excluded, the high- and low-performance subgroups were similarly distributed by sex/gender (high: *n* = 220, 53 cisgender men [24.1%], 167 cisgender women [75.9%]; low: *n* = 139, 23 cisgender men [16.5%], 116 cisgender women [83.5%]).

We examined the effect of sex/gender, feedback condition, and feedback accuracy on feedback credibility using a 2 (cisgender men, cisgender women) × 2 (feedback condition: positive, negative) × 2 (feedback accuracy: true, false) univariate ANOVA on the reduced sample described above (see Credibility of Feedback). Significance level for post-hoc tests was set at *p* < .01.

A main effect of feedback accuracy emerged, *F*(1, 348) = 7.62, *p* = .006, *η*_p_^2^ = .02, such that participants who received true feedback rated it as more credible (3.80 ± 1.89) than those who received false feedback (3.10 ± 1.69). There was also a significant interaction between feedback valence and accuracy, *F*(1, 348) = 5.24, *p* = .023, *η*_p_^2^ = .02.

Follow-up one-way ANOVAs indicated that credibility ratings did not differ significantly between high performers who received true positive feedback (3.42 ± 1.90) and low performers who received false positive feedback (3.03 ± 1.82), *F*(1, 180) = 1.90, *p* = .170, *η*_*p*_^2^ = .01. In contrast, low performers rated true negative feedback (4.45 ± 1.71) as substantially more credible than high performers rated false negative feedback (3.16 ± 1.61), *F*(1, 172) = 25.00, *p* < .001, *η*_*p*_^2^ = .13. Furthermore, low performers perceived true negative feedback as more credible than false positive feedback, *F*(1, 135) = 21.88, *p* < .001, *η*_*p*_^2^ = .14, whereas high performers rated feedback similarly regardless of accuracy (*p* = .271). No other main effects or interactions approached significance (all *F* < 2.62, *p* > .106).

Given these findings, feedback accuracy was retained as a factor in all subsequent analyses.

### Mental Rotation Performance

We examined the effect of sex/gender, timepoint, feedback condition, and feedback accuracy on MRT performance using a 2 (cisgender men, cisgender women) × 2 (T1, T2) × 3 (feedback condition: positive, negative, none) × 3 (feedback accuracy: true, false, no feedback) mixed ANOVA. Significance level for post-hoc tests was set at *p* < .01.

A significant main effect of sex/gender emerged, *F*(1, 452) = 6.67, *p* = .010, *η*_*p*_^*2*^ = .02, with cisgender men (5.87 ± 2.65), outperforming cisgender women (4.97 ± 2.51). There was also a significant main effect of timepoint, *F*(1, 452) = 30.31, *p* < .001, *η*_*p*_^*2*^ = .06, with higher MRT scores at T2 (5.45 ± 3.02) than T1 (4.87 ± 2.80).

A feedback condition × feedback accuracy interaction was significant, *F*(1, 452) = 133.04, *p* < .001, *η*_*p*_^*2*^ = .23. Post-hoc one-way ANOVAs indicated that participants receiving true positive feedback (6.67 ± 2.15) scored significantly higher than those receiving false positive feedback (3.02 ± 1.38), *F*(1, 181) = 164.64, *p* < .001, *η*_*p*_^*2*^ = .48. Similarly, those receiving false negative feedback (6.32 ± 2.07) outperformed those receiving true negative feedback (3.08 ± 1.19), *F*(1, 174) = 134.56, *p* < .001, *η*_*p*_^*2*^ = .44. These differences reflect pre-existing performance disparities between high (who received true positive or false negative feedback) and low performers (who received false positive or true negative feedback) which persisted regardless of feedback.

The three-way interaction of timepoint × feedback condition × feedback accuracy was also significant, *F*(1, 452) = 37.42, *p* < .001, *η*_*p*_^*2*^ = .08. Post-hoc paired *t*-tests showed significantly higher performance at T2 (3.81 ± 2.58) than T1 (2.23 ± 0.89) for low performers receiving false positive feedback, *t*(72) = -4.99, *p* < .001, *d* = -0.58, and for low performers receiving true negative feedback (T1: 2.09 ± 0.92, T2: 4.08 ± 2.14), *t*(65) = -7.02, *p* < .001, *d* = -0.86. No significant timepoint effects were observed among high performers in any feedback condition or in the control condition, all *p*s > .509.

**Fig. 2:**
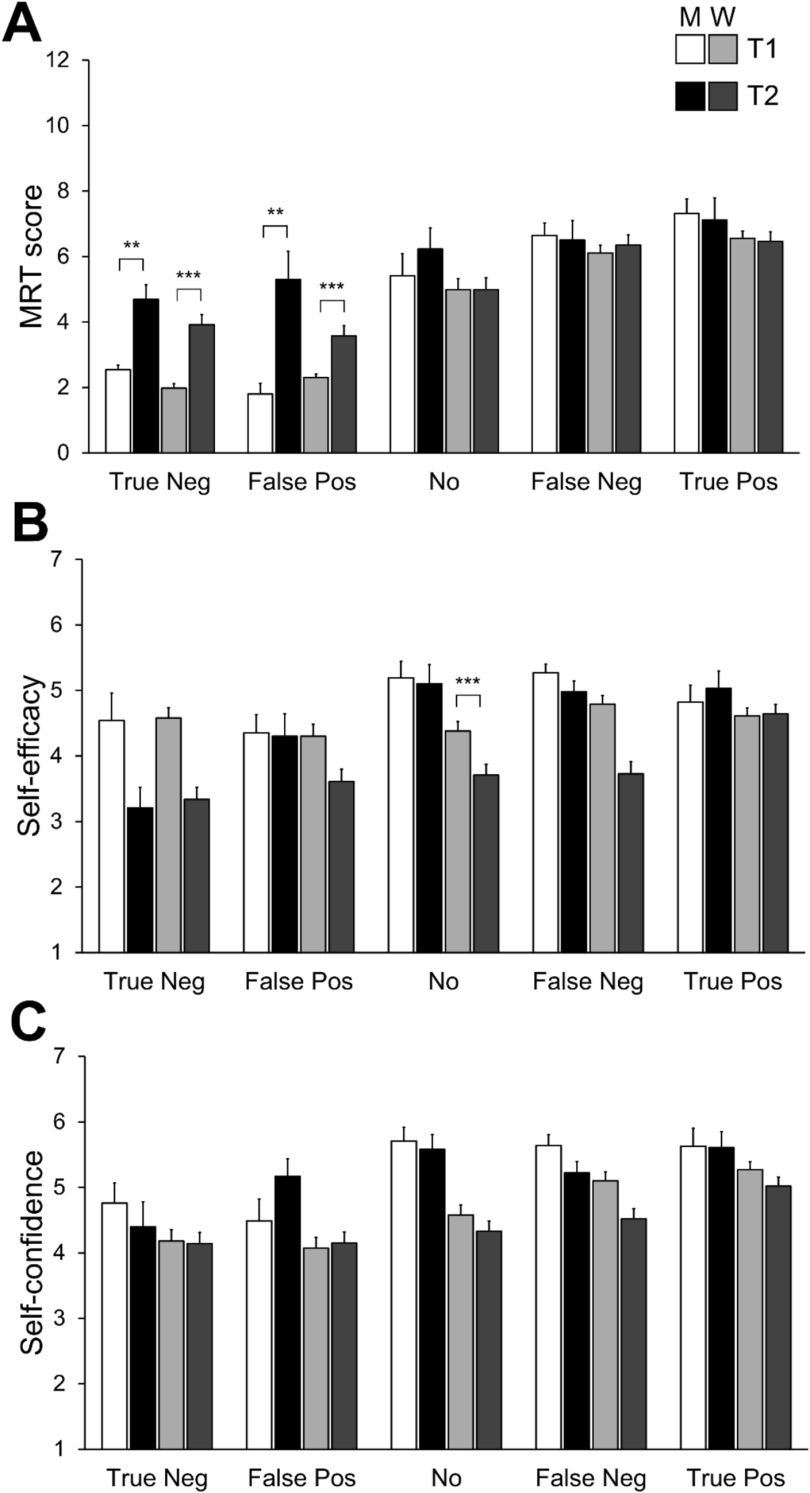
Effect of timepoint, sex/gender, feedback condition, and feedback accuracy on mean (A) MRT scores, (B) mental rotation self-efficacy, and (C) self-confidence in the MRT in the true negative (True Neg), false positive (False Pos), no feedback (No), false negative (False Neg) and true positive feedback (True Pos) conditions. Error bars represent standard error. White (T1) and black (T2) bars represent cisgender men. Light gray (T1) and dark gray (T2) bars represent cisgender women. **p < .01, ***p < .001

The four-way interaction of sex/gender × timepoint × feedback condition × feedback accuracy was also significant, *F*(1, 452) = 3.92, *p* = .048, *η*_*p*_^*2*^ = .01. Post-hoc paired *t*-tests revealed significant T2 performance gains among low-performing cisgender men (false positive: T1: 1.80 ± 1.03, T2: 5.30 ± 2.71, *t*(9) =  − 4.27, *p* = .002, *d* = − 1.35; true negative: T1: 2.54 ± 0.52, T2: 4.69 ± 1.60, *t*(12) = − 4.07, *p* = .002, *d* = − 1.13) and low-performing cisgender women (false positive: T1: 2.30 ± 0.85, T2: 3.57 ± 2.50, *t*(62) =  − 3.87, *p* < .001, *d* = − 0.49; true negative: T1: 1.98 ± 0.97, T2: 3.92 ± 2.24, *t*(52) = − 5.90, *p* < .001, *d* = − 0.81). No significant effects were found for high performers, regardless of feedback type or sex/gender, or for participants in the control condition, all *p*s > .127. Post-hoc *t*-tests assessing sex/gender differences within each feedback and timepoint condition were non-significant, all *p*s > .048. No other main effects or interactions approached significance (all *F* < 3.30, *p* > .070; see Fig. 2).

### Self-Perception of Spatial Abilities

#### Discriminant Validity

Because spatial anxiety, self-efficacy in spatial abilities, and MRT self-confidence are often interrelated (e.g., Arrighi & Hausmann, 2022), and conceptual overlap was expected, particularly between spatial anxiety and self-efficacy, we conducted exploratory analyses to assess discriminant validity among these constructs.

Bivariate correlations among T1 measures revealed that, although all correlations were significant (*p*s < .001), none exceeded *r* > 0.70, suggesting adequate distinction between scales.

An exploratory factor analysis (EFA) was then conducted on all 36 items from the T1 measures (spatial anxiety: SA1-SA12; self-efficacy: SE1-SE12; self-confidence: SC1-SC12) using maximum likelihood extraction and direct oblimin rotation (minimum factor loading: 0.40, eigenvalue cutoff of 1). Sampling adequacy was acceptable, χ^2^(630) = 2298.61, *p* < .001, Kaiser–Meyer–Olkin [KMO] = 0.80. The initial EFA yielded six factors explaining 60.46% of the variance. However, five items (SA4, SA6, SA8, SA10, SA11) did not load on any factors, four items (SA7, SA12, SE3, SE8) exhibited cross-loadings > 0.40, and five items (SA1, SA3, SA5, SE2, SE6) had low loadings (< 0.50) and were removed.

The second EFA (KMO = 0.88) identified three factors explaining 61.89% of the variance, but two items (SA2, SA9) failed to meet loading criteria and were removed.

The final EFA (KMO = 0.90) produced three factors explaining 65.28% of the variance, with all communalities > 0.45. Factor 1 comprised all self-confidence questions. Factor 2 included self-efficacy items specifically referencing mental rotation (SE9-SE12). Factor 3 included self-efficacy items related to navigation and wayfinding (SE1, SE4, SE5, SE7). No spatial anxiety items loaded onto this final factorial structure and were therefore removed from all subsequent analyses. Rotated factor loadings are presented in Table 2.

**Table 2:** Rotated factor loadings

Item	Factor 1	Factor 2	Factor 3
SC1	0.82	-	-
SC2	0.87	-	-
SC3	0.82	-	-
SC4	0.85	-	-
SC5	0.83	-	-
SC6	0.88	-	-
SC7	0.89	-	-
SC8	0.85	-	-
SC9	0.84	-	-
SC10	0.68	-	-
SC11	0.77	-	-
SC12	0.81	-	-
SE1	-	-	0.79
SE4	-	-	0.62
SE5	-	-	0.57
SE7	-	-	0.70
SE9	-	0.85	-
SE10	-	0.85	-
SE11	-	0.68	-
SE12	-	0.63	-

A confirmatory factor analysis (CFA) in AMOS v29 with the items in Table 2 supported the EFA structure, although covariance between Factors 2 and 3 exceeded 0.50, indicating insufficient discriminant validity. The CFA was therefore repeated with only Factors 1 and 2. In this model, all standardized loadings exceeded 0.70 except SC10 (0.62), and factor covariance was 0.43. Model fit was acceptable: χ^2^(103) = 256.63, *p* < .001, CMIN/DF = 2.49, NFI = 0.93, CFI = 0.96, RMSEA = 0.056 (90% CI: 0.048–0.065, PCLOSE = 0.116). The SRMR could not be computed due to incomplete self-confidence data. Overall, the indices supported the independent validity of self-confidence (Factor 1) and self-efficacy in mental rotation (Factor 2). For subsequent analyses, self-confidence was retained as originally measured (see Measures: Self-confidence in the MRT). A new variable, mental rotation self-efficacy, was computed based on Factor 2 (items SE9-SE12; see Measures: Self-efficacy in Spatial Abilities). At T1, both self-confidence and mental rotation self-efficacy were significantly correlated with average MRT performance (self-confidence: *r*(461) = 0.52,* p* < .001; mental rotation self-efficacy: *r*(462) = 0.23,* p* < .001) as well as with one another (*r*(461) = 0.38,* p* < .001). As none of the spatial anxiety items met the loading criteria, the spatial anxiety measure was excluded from all subsequent analyses.

#### Self-Efficacy in Mental Rotation

We examined the effects of sex/gender, timepoint, feedback condition, and feedback accuracy on mental rotation self-efficacy using a 2 (cisgender men, cisgender women) × 2 (T1, T2) × 3 (positive, negative, no feedback) × 3 (true, false, no feedback) mixed ANOVA. Significance level for post-hoc tests was set at *p* < .01.

The main effect of sex/gender was significant, *F*(1, 452) = 16.33, *p* < .001, *η*_*p*_^*2*^ = .04, with cisgender men (4.83 ± 1.12) reporting higher self-efficacy than cisgender women (4.20 ± 1.24). The main effect of timepoint was also significant, *F*(1, 452) = 51.05, *p* < .001, *η*_*p*_^*2*^ = 0.10, with lower self-efficacy at T2 (4.04 ± 1.53) than T1 (4.63 ± 1.23).

The timepoint × sex/gender interaction was significant, *F*(1, 452) = 9.72, *p* = .002, *η*_*p*_^*2*^ = 0.02. Post-hoc paired *t*-tests indicated a significant decline in self-efficacy from T1 (4.54 ± 1.24) to T2 (3.86 ± 1.53) in cisgender women, *t*(363) = 10.14, *p* < .001, *d* = 0.53, but no significant change for cisgender men, *p* = .030. A timepoint × feedback condition interaction was also significant, *F*(1, 452) = 28.08, *p* < .001, *η*_*p*_^*2*^ = .06. Self-efficacy declined significantly from T1 to T2 in the negative feedback condition (T1: 4.79 ± 1.15, T2: 3.77 ± 1.52), *t*(175) = 11.07, *p* < .001, *d* = 0.83), and in the control condition albeit with a smaller effect size (T1: 4.55 ± 1.32, T2: 4.00 ± 1.55), *t*(102) = 4.67, *p* < .001, *d* = 0.46). No significant change was observed in the positive feedback condition, *p* = .026. The feedback condition × feedback accuracy interaction was also significant, *F*(1, 452) = 18.12, *p* < .001, *η*_*p*_^*2*^ = .04. Within the positive feedback condition, self-efficacy was higher among participants receiving true feedback (4.69 ± 1.15) compared to those receiving false positive feedback (4.01 ± 1.24), *F*(1, 181) = 14.36, *p* < .001, *η*_*p*_^*2*^ = .07). Within the negative feedback condition, self-efficacy was higher for false feedback (4.48 ± 1.22) than for true feedback (3.95 ± 1.10), *F*(1, 174) = 8.55, *p* = .004, *η*_*p*_^*2*^ = .05. These disparities, similar to those found in MRT performance, reflect pre-existing self-efficacy disparities between high (who received true positive or false negative feedback) and low performers (who received false positive or true negative feedback) which persisted regardless of the feedback received.

The three-way interaction of timepoint × feedback condition × feedback accuracy was also significant, *F*(1, 452) = 11.61, *p* < .001, *η*_*p*_^*2*^ = .03. Declines from T1 to T2 were observed for those who received false negative (T1: 4.91 ± 1.09, T2: 4.05 ± 1.57), true negative (T1: 4.58 ± 1.21, T2: 3.31 ± 1.31), and false positive feedback (T1: 4.31 ± 1.38, T2: 3.71 ± 1.46), all *p*s < .001, with medium-to-large effect sizes (*d*s = 0.73, 1.02, and 0.47, respectively). No significant changes were found following true positive feedback, *p* = .449.

The timepoint × sex/gender × feedback accuracy interaction was also significant, *F*(1, 452) = 4.22, *p* = .041, *η*_*p*_^*2*^ = .01. Cisgender women showed significant declines in self-efficacy regardless of feedback accuracy or control condition (all *p*s < .001, *d*s = 0.35–0.72), whereas no significant effect were found for cisgender men, *p*s > .128. Sex/gender differences favoring cisgender men emerged at T2 in the false feedback condition (*p* < .001, *d* = 0.77) and were present at both timepoints in the control condition (*p*s ≤ .010, *d*s = 0.64–0.96). No sex/gender differences were observed in the true feedback condition at either timepoint (*p*s > .332). No other main effect or interaction approached significance (all *F* < 2.39, *p* > .122; see Fig. 2).

#### Self-Confidence in the MRT

We next examined the effects of sex/gender, timepoint, feedback condition, and feedback accuracy on self-confidence in the MRT using the same 2 × 2 × 3 × 3 mixed ANOVA. Significance level for post-hoc tests was again set at *p* < .01.

The main effect of sex/gender was significant, *F*(1, 447) = 27.77, *p* < .001, *η*_*p*_^*2*^ = .06 with cisgender men (5.35 ± 1.06) reporting higher self-confidence than cisgender women (4.59 ± 1.26). The main effect of timepoint was also significant, *F*(1, 447) = 6.36, *p* = .012, *η*_*p*_^*2*^ = .01, with lower self-confidence at T2 (4.65 ± 1.35) than T1 (4.86 ± 1.32).

The timepoint × feedback condition interaction was significant, *F*(1, 447) = 14.61, *p* < .001, *η*_*p*_^*2*^ = 0.03. Self-confidence declined from T1 to T2 in the negative feedback condition (T1: 4.89 ± 1.28, T2: 4.51 ± 1.31, *t*(173) = 5.01, *p* < .001, *d* = 0.38), and marginally in the control condition (T1: 4.83 ± 1.34, T2: 4.60 ± 1.39, *t*(100) = 2.50, *p* = .014, *d* = 0.25). No significant change occurred in the positive feedback condition, *p* = .374.

The feedback condition × feedback accuracy interaction was also significant, *F*(1, 447) = 26.05, *p* < .001, *η*_*p*_^*2*^ = .06. In the positive feedback condition, self-confidence was higher for true (5.26 ± 1.17) than for false feedback (4.21 ± 1.24), *F*(1, 180) = 33.48, *p* < .001, *η*_*p*_^*2*^ = .16. In the negative feedback condition, self-confidence was higher for false (4.97 ± 1.17) than for true feedback (4.25 ± 1.12), *F*(1, 172) = 16.10, *p* < .001, *η*_*p*_^*2*^ = .09. These disparities, similar to those found in MRT performance, reflect pre-existing self-confidence disparities between high (who received true positive or false negative feedback) and low performers (who received false positive or true negative feedback) which persisted regardless of the feedback received.

The timepoint × feedback condition × feedback accuracy interaction was significant, *F*(1, 447) = 10.60, *p* = .001, *η*_*p*_^*2*^ = .02. Declines from T1 to T2 were observed following true positive and false negative feedback (*p*s ≤ .006, *d*s = 0.27–0.63) but not following false positive or true negative feedback (*p*s > .129).

A timepoint × sex/gender × feedback condition interaction was also found, *F*(1, 447) = 4.03, *p* = .045, *η*_*p*_^*2*^ = .01. Declines from T1 to T2 occurred for cisgender men and women in the negative feedback condition (*p*s ≤ .004, *d*s = 0.36–0.48) and marginally for cisgender women in the control condition (*p* = .016, *d* = 0.28), but not for those receiving positive feedback or for cisgender men in the control condition (*p*s > .102).

Post-hoc comparisons revealed that sex/gender differences in self-confidence emerged at T2 in the positive feedback condition (*p* < .001, *d* = 0.64) and were present at both timepoints in the negative feedback and control conditions (*p*s < .011). No other main effects or interactions approached significance (all *F* < 2.90, *p* > .089; see Fig. 2).

### Mediation Analysis

To investigate whether the effects of feedback on MRT performance were direct or mediated by changes in mental rotation self-efficacy and self-confidence (Hypothesis 5), we conducted an exploratory parallel mediation analysis on the subset of low performers (*n* = 139) who had shown significant performance gains from T1 to T2. Participants in the control condition (*n* = 103) served as reference group. Three participants with incomplete self-confidence data were excluded, resulting in a total sample of *n* = 239.

**Fig. 3:**
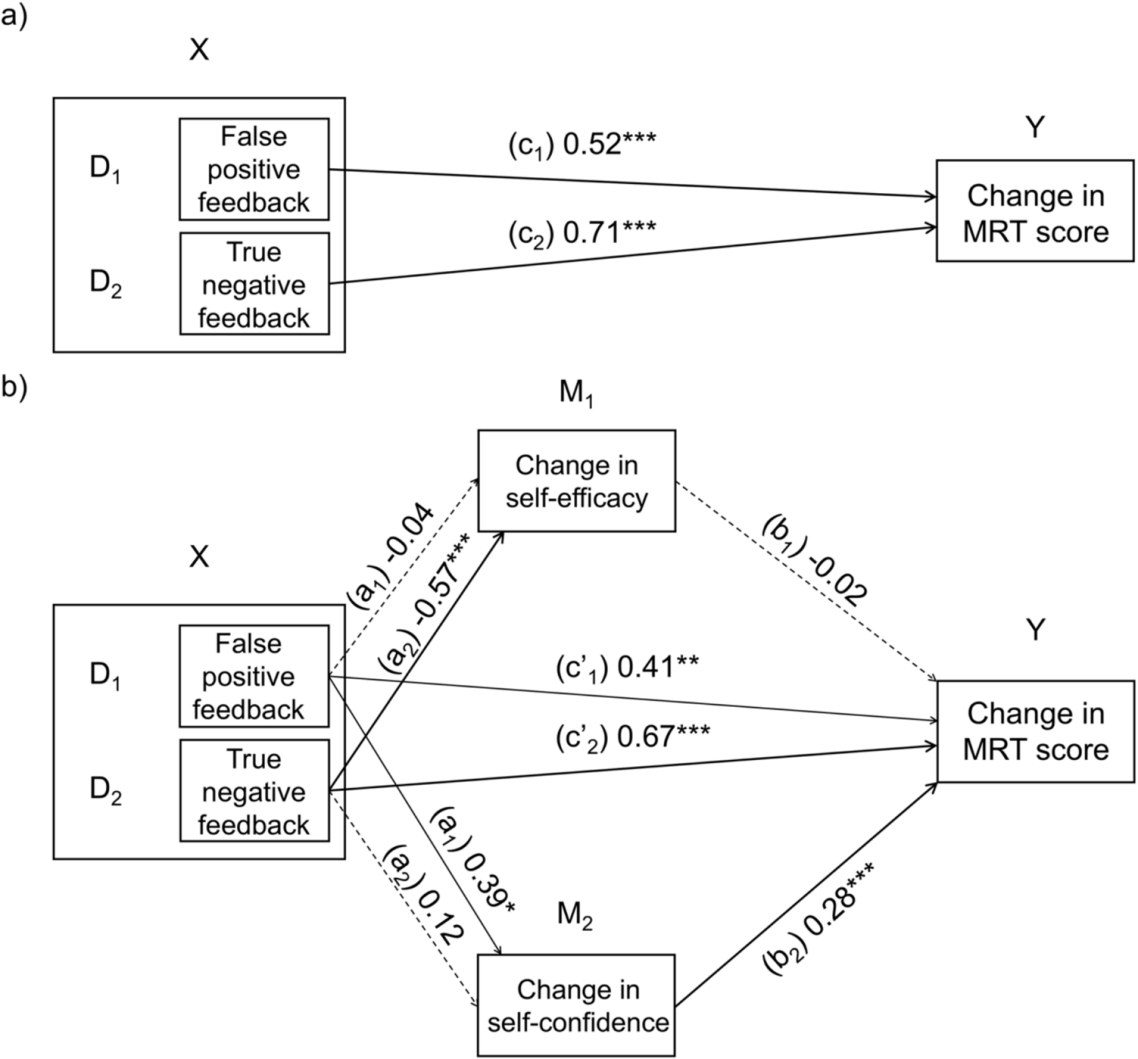
Total, direct, and indirect effects of feedback on MRT score via self-efficacy and self-confidence. The figure is a schematic representation of a parallel mediation model with a multi-categorical independent variable (feedback condition, with 3 categories) and two mediators, change in self-efficacy (M_1_) and change in self-confidence (M_2_), through which feedback condition (X) exerts its effect on MRT score change (Y). M_1,_ M_2_, and Y are standardized. D_1_ codes the false positive feedback condition, D_2_ codes the true negative feedback condition, and the control condition functions as the reference group and receives a code of 0 on D_1_ and D_2_. The total effect model (model a), or c path) was significant. Coefficients are standardized. N = 239. *p < .05, **p < .01, ***p < .001

The model was tested using the PROCESS macro for SPSS v5.0 beta (Model 4 adapted for a multi-categorical independent variable; Hayes, 2012), with feedback condition as the multi-categorical independent variable, standardized change scores in self-efficacy and self-confidence from T1 to T2 as parallel mediators, and standardized change in MRT performance as the dependent variable (see Fig. 3). The control condition (no feedback) served as the reference category. Sex/gender was not included, as the feedback-performance relationship was similar for cisgender men and women (see Fig. 2). Bias-corrected confidence intervals (95%) were generated from 10,000 bootstrap samples, stratified by group. Mediation was inferred when CI did not include zero.

The total effect of feedback condition on MRT change was significant, *R*^*2*^ = 0.10, *F*(2, 236) = 12.48, *p* < .001. Both false positive (D_1_) and true negative feedback (D_2_) were associated with greater improvement compared to the control condition: c_1_ = 0.52, *SE* = 0.15, *t*(2, 236) = 3.57, *p* < .001, 95% CI [0.24, 0.81]; c_2_ = 0.71, *SE* = 0.15, *t*(2, 236) = 4.64, *p* < .001, 95% CI [0.41, 1.00].

Including the two mediators increased explained variance by 7% (*R*^*2*^ = 0.17, *∆R*^*2*^ = 0.07, *F*(4, 234) = 11.89, *p* < .001). The direct effects remained significant but were smaller than the total effects: false positive feedback, c′_1_ = 0.41, *SE* = 0.14, *t*(4, 234) = 2.90, *p* = .004, 95% CI [0.14, 0.69]; true negative, c′_2_ = 0.67, *SE* = 0.15, *t*(4, 234) = 4.40, *p* < .001, 95% CI [0.38, 0.95], indicating a partial mediation.

For the indirect paths, false positive feedback was not associated with changes in self-efficacy, a_1_ = − 0.04, *SE* = 0.15, *t*(2, 236) = 0.25, *p* = .799, 95% CI [− 0.34, 0.26], whereas true negative feedback predicted decreases in self-efficacy, a_2_ = − 0.57, *SE* = 0.15, *t*(2, 236) = -3.64, *p* < .001, 95% CI [− 0.87, − 0.27]. Conversely, false positive feedback predicted increases in self-confidence, a_1_ = 0.39, *SE* = 0.14, *t*(2, 236) = 2.58, *p* = .010, 95% CI [0.12, 0.68], while true negative feedback had no significant effect, a_2_ = 0.12, *SE* = 0.12, *t*(2, 236) = 0.77, *p* = .443, 95% CI [− 0.21, 0.47].

Changes in self-efficacy were unrelated to MRT change, b_1_ = − 0.02, *SE* = 0.06, *t*(4, 234) =  − 0.25, *p* = .805, 95% CI [− 0.13, 0.10], but increases in self-confidence predicted greater MRT gains, b_2_ = 0.28, *SE* = 0.07, *t*(4, 234) = 4.54, *p* < .001, 95% CI [0.14, 0.41]. The only significant indirect effect was that of false positive feedback via self-confidence, a_1_b_2_ = 0.11, *SE* = 0.05, 95% CI [0.03, 0.22]. Thus, performance benefits of false positive feedback were partly mediated by increases in self-confidence, whereas the benefits of true negative feedback appeared to operate through a direct pathway, independent of changes in self-confidence or self-efficacy.

## Discussion

The present study directly manipulated participants’ beliefs about their own spatial abilities by providing randomized normative feedback, to examine whether such manipulation would influence cognitive performance and/or the size of sex/gender differences in MRT scores. Consistent with prior research, the findings replicated the well-documented sex/gender difference in MRT performance (Hypothesis 1). Specifically, cisgender men outperformed cisgender women, with an effect size (*d* = 0.35) at the lower end of the range reported in previous meta-analyses (e.g., Linn & Petersen, 1985; Reilly & Neumann, 2013; Voyer et al., 1995). This comparatively smaller effect may be attributable to characteristics of the current sample, such as a relatively high proportion of participants identifying as sexual orientation diverse (25%; see Table 1). Indeed, existing research suggests that sex/gender differences in mental rotation tasks tend to be smaller in sexual orientation diverse samples compared to predominantly heterosexual ones (Xu et al., 2020).

Moreover, societal shifts over the past two decades toward greater awareness of sex and gender diversity (Hammack & Wignall, 2023) complicate direct comparisons with earlier studies, which often failed to examine participants' sex-/gender-related characteristics and views on sex/gender, which have been found to affect sex/gender differences in the MRT (e.g., pronouncedness of sex/gender stereotypes was related to MRT performance in two previous studies: Hausmann et al., 2009; Moè et al., 2021). Given that both sample composition and evolving conceptualizations of sex/gender can influence the size of sex/gender differences, these factors must be carefully accounted for in future research assessing cognitive sex/gender differences.

In addition to the significant sex/gender differences observed in MRT performance, we also found the anticipated sex/gender disparities in mental rotation self-efficacy and task-specific self-confidence, with cisgender men reporting higher levels than cisgender women in both constructs (self-efficacy in mental rotation: *d* = 0.52; self-confidence: *d* = 0.62; Hypothesis 1). These findings and effect sizes align with prior research examining related constructs, such as spatial anxiety (Alvarez-Vargas et al., 2020), self-confidence (Cooke-Simpson & Voyer, 2007; Estes & Felker, 2012), self-efficacy (Miola et al., 2021; Towle et al., 2005), and self-reported spatial abilities (Matthews et al., 2024, meta-analysis). Although self-efficacy and self-confidence emerged as distinct factors, spatial anxiety items showed low or cross-loadings and were removed. This indicates spatial anxiety was insufficiently distinct from self-efficacy and self-confidence and/or its items lacked coherence. One explanation may be that existing measures of domain-specific anxieties typically assess how anxious certain situations would make participants feel (e.g., Lyons et al., 2018; Maloney et al., 2015), whereas we asked participants how much they identified with statements such as “I am generally secure when solving spatial problems” probing trait rather than state spatial anxiety. Without explicit prompts to consider situational anxiety, participants may have instead focused on item wording or the situations themselves—leading to less consistency of interpretation across items and participants. Although spatial anxiety was excluded from analyses, we assume the current findings may also apply to this construct, since—similar to self-efficacy and self-confidence—it has been shown to be sex/gender-sensitive (Lyons et al., 2018) and correlate with mental rotation performance (Alvarez-Vargas et al., 2020; Arrighi & Hausmann, 2022).

Notably, our results revealed that cisgender women (but not cisgender men) demonstrated a decline in both self-confidence and self-efficacy following the completion of the mental rotation task in the control condition, despite no observable change in actual performance across timepoints. This pattern mirrors earlier findings in other cognitive domains, such as self-confidence in creative abilities (McCarty, 1986), and aligns with hypotheses outlined in seminal reviews on gendered self-perceptions (Lenney, 1977). Supporting our results, recent studies have shown that women tend to underestimate their spatial abilities, whereas men provide more accurate self-assessments (Hofer et al., 2025), and that women are also more likely to underestimate their intelligence relative to men, even in the absence of sex/gender differences in objective performance (e.g., Reilly et al., 2022). This phenomenon is often referred to as the male hubris, female humility effect. Future research should explore the mechanisms underlying this decline in self-beliefs among cisgender women in greater depth, particularly in contexts involving cognitive performance assessments.

The central aim of the present study was to experimentally manipulate participants’ beliefs in their spatial abilities by providing randomized normative feedback. Notably, participants generally exhibited low levels of trust in the feedback, with true negative feedback perceived as the most credible and false positive feedback as the least credible. Nevertheless, feedback significantly influenced participants’ self-beliefs. As hypothesized (Hypothesis 3), negative feedback, regardless of its accuracy, was associated with a reduction is spatial self-efficacy and self-confidence among both cisgender men and women. This outcome aligns with motivation theory, which posits that negative feedback can diminish both motivation and self-perception (Ryan & Deci, 2000).

Additionally, as previously noted, even the absence of feedback led to decline in spatial self-perceptions among cisgender women, but not among cisgender men. These results suggest that both negative feedback and lack of feedback can adversely affect individuals’ beliefs in their spatial abilities, with particularly pronounced effects for cisgender women. Similar findings were reported by McCarty (1986), who showed that receiving no feedback significantly reduced self-confidence in creativity across both sexes/genders. One possible explanation for these effects lies in the difficulty of the task. Prior research has shown that the MRT often yields lower performance scores compared to other spatial tasks (Collins & Kimura, 1997; Hausmann et al., 2009), indicating that it is particularly challenging. This perception of difficulty appears to have been shared by participants in the current study, as MRT-specific self-confidence ratings were numerically lower in both cisgender men (5.35 ± 1.06) and women (4.59 ± 1.26) compared to those reported in previous studies (men: 6.14 ± 0.77, women: 4.99 ± 1.11; e.g., Cooke-Simpson & Voyer, 2007). It is therefore plausible that merely completing a difficult cognitive task, such as the MRT, may prompt participants, and especially cisgender women, to adjust their beliefs about their spatial abilities downward, even in the absence of negative feedback.

The effects of positive feedback on self-beliefs were generally comparable across cisgender men and women but differed depending on the accuracy of the feedback. Specifically, when positive feedback was accurate (i.e., true), participants’ self-confidence decreased, while self-efficacy remained unchanged. In contrast, when the positive feedback was inaccurate (i.e., false), it was associated with an increase in self-confidence (as indicated by the mediation analysis), but a decrease in self-efficacy. These divergent effects suggest that the impact of positive feedback on self-perception is not straightforward and may depend on participants’ internal evaluations of their own performance relative to the feedback received.

The lack of correspondence between changes observed across different self-perception variables may be explained by the temporal dynamics of the feedback effects, as described by Peifer et al. (2020). In their study, participants received randomized normative positive feedback after completing the first of two sets of an arithmetic task, and self-efficacy was measured at three different timepoints. While an immediate increase in self-efficacy was observed post-feedback, this effect diminished by the end of the second set, returning to baseline levels. Although speculative, similar temporal fluctuations may have occurred in the present study, with participants initially experiencing a confidence boost (or decrease) that was not sustained over time. Future research should systematically investigate the time course of feedback effects on both self-confidence and self-efficacy, particularly in challenging (spatial) cognitive domains.

We had anticipated that feedback would produce sex-/gender-specific effects on spatial self-perception. However, contrary to Hypothesis 4, and despite the robust baseline sex/gender differences in spatial self-beliefs, feedback influenced self-confidence similarly in cisgender men and women. This finding is consistent with prior studies using both a chronometric mental rotation task (Rahe et al., 2019) and a creativity task (McCarty, 1986) which likewise found no significant sex/gender differences in the effects of feedback on perceived task difficulty or self-confidence.

Nonetheless, sex-/gender-specific effects did emerge for mental rotation self-efficacy. Cisgender women were more likely than cisgender men to exhibit reduced self-efficacy following the task, regardless of the accuracy or presence of feedback. This suggests that self-efficacy in spatial domains may be particularly sensitive to external evaluative cues in cisgender women, reinforcing prior findings that women tend to underestimate their (spatial) abilities (e.g., Hofer et al., 2025; Matthews et al., 2024). Taken together, these results underscore the importance of considering sex-/gender-related patterns not only in baseline self-beliefs but also in how individuals internalize and respond to performance feedback.

While feedback significantly altered participants’ self-perception of spatial abilities, these changes did not consistently translate into corresponding changes in (spatial) cognitive performance (Hypotheses 2–3). Specifically, for conditions involving true positive, false negative, and no feedback, declines in self-perception were not accompanied by changes in performance. Interestingly, true negative feedback was associated with decreased self-efficacy and improved (spatial) cognitive performance, whereas false positive feedback led to increased self-confidence, reduced self-efficacy, and a concurrent improvement in performance. These findings partly diverge from prior studies suggesting that feedback, whether accurate (Rahe et al., 2019) or randomized positive (Estes & Felker, 2012), can enhance mental rotation performance in terms of both accuracy and reaction time.

One important methodological distinction lies in how feedback was implemented and evaluated. Previous studies compared between feedback types (e.g., positive vs. negative, Estes & Felker, 2012; and accurate vs. no feedback, Rahe et al., 2019), without considering whether the feedback reflected participants’ actual baseline performance or assessing credibility of the feedback. The present study addressed both issues by comparing pre- and post-feedback performance and measuring participants’ trust in the feedback provided. These methodological improvements may help explain the discrepancies with earlier findings and raise important questions about the role of perceived feedback credibility. Additionally, previous studies did not control for possible pre-existing group differences, leaving open the possibility that observed feedback effects were confounded by baseline disparities in spatial performance.

Another plausible explanation for the absence of performance effects following true positive and false negative feedback may be participants’ low trust in the feedback. This points to the need for future research to explore alternative feedback strategies that may be more credible, and hence effective in manipulating self-beliefs and performance. For instance, providing detailed feedback (e.g., highlighting correct answers or offering task-specific guidance) or formative feedback (i.e., practical task-solving tips) has been found to enhance performance more reliably than uninformative evaluative feedback (Kluger & DeNisi, 1996; Shute, 2008; Wisniewski et al., 2020). A particular promising direction may involve providing all participants with accurate, item-level feedback while simultaneously measuring perceived feedback credibility at multiple time points.

As noted earlier, the effects of feedback on self-perceptions were not always mirrored by changes in (spatial) cognitive performance. This asymmetry suggests that the causal relationship between self-perception and performance may be unidirectional, flowing from performance to self-perception, rather than vice versa. Our findings suggest that altering self-beliefs alone is neither necessary nor sufficient to elicit cognitive performance changes. A similar conclusion was reached by a systematic review by Balt et al. (2022) comparing interventions targeting math anxiety: interventions that improved math performance also reduced anxiety, but interventions that reduced anxiety did not necessarily improve performance. Although a unidirectional pathway seems plausible, the observed effects of false positive feedback, where both self-perception and performance improved, suggest that they may have a common underlying factor, such as general self-concept or motivation. Future research should further disentangle these relationships to clarify the causal dynamics between self-beliefs and cognitive performance and identify potential shared latent factors.

Given the well-documented sex/gender differences in MRT performance, we hypothesized that feedback would show sex-/gender-specific effects on performance. Contrary to Hypothesis 4, feedback influenced (spatial) cognitive performance similarly across cisgender men and women. This aligns with previous findings (Estes & Felker, 2012) suggesting that performance feedback does not differentially affect cognitive performance based on sex/gender. However, emerging evidence indicates that other forms of feedback, such as encouragement, may have sex-/gender-specific effects on cognitive performance. For example, Lovász et al. (2022) found that encouragement improved women’s, but not men’s, performance on a visual perception task. Future studies may benefit from examining the potential of encouragement-based interventions (e.g., Lovász et al., 2022; Unkovic et al., 2016), particularly given the consistent findings that women tend to report lower confidence in their spatial abilities (Arrighi & Hausmann, 2022; Cooke-Simpson & Voyer, 2007; Estes & Felker, 2012).

To further explore the performance improvements observed in low performers receiving either true negative or false positive feedback, we conducted a parallel mediation analysis (Fig. 3). The analysis revealed that the effect of false positive feedback on (spatial) cognitive performance was partially mediated by increases in self-confidence (Hypothesis 5). Notably, this effect occurred despite participants generally rating the feedback as low in credibility. This finding is consistent with prior studies showing that false positive feedback can boost self-efficacy and performance in tasks unrelated to spatial cognition (Grealy et al., 2019; Vongjaturapat, 1992). The findings also align with *motivation theory* (Ryan & Deci, 2000), which posits that positive feedback enhances performance by increasing self-beliefs and motivation. It is plausible that false positive feedback improved both self-confidence and performance by enhancing participants’ motivation—a hypothesis aligned with a recent review proposing that motivation may serve as a key mediator between self-beliefs and (spatial) cognitive performance (Lourenco & Liu, 2023).

In contrast, for participants receiving true negative feedback, (spatial) cognitive performance also improved, but without a corresponding increase in self-beliefs. Given that this group rated the feedback as more credible, the performance gain is unlikely to stem from distrust in the feedback. A possible explanation lies in the literature on stress and performance. Negative feedback may have triggered a moderate stress response, which, under certain conditions, can enhance rather than impair cognitive functioning (Cohen et al., 2020; Salehi et al., 2010; Yerkes & Dodson, 1908). Additionally, it is possible that participants were effective emotional reappraisers. Raftery and Bizer (2009) found that negative feedback improved performance in a visuospatial task, but only among individuals who habitually reappraised emotional situations rather suppressing them. The absence of improvements in self-confidence, and the simultaneous decline in self-efficacy, remain surprising, especially considering the well-established buffering effect of self-efficacy under stress (Bandura, 1995; Fida et al., 2015; Gallagher et al., 2020; O’Leary, 1992). It is possible that performance-enhancing and self-belief-diminishing effects of true negative feedback operated through distinct mechanisms, which may explain the lack of a mediation effect in our model.

### Limitations

One limitation of the present study is the unequal group sizes, with fewer cisgender men than cisgender women. This imbalance may have limited the statistical power to detect effects in cisgender men and led to an overrepresentation of cisgender women in the overall findings. Nevertheless, the cisgender men and women in this sample were comparable in demographic characteristics, and the sex/gender differences observed across variables closely mirror those reported in prior research (Alvarez-Vargas et al., 2020; Arrighi & Hausmann, 2022; Cooke-Simpson & Voyer, 2007; Estes & Felker, 2012; Lauer et al., 2018; Lourenco & Liu, 2023; Malanchini et al., 2017; Matthews et al., 2024), supporting the representativeness of the male sample.

Another key limitation is the exclusive focus on cisgender participants, which precludes a nuanced analysis of the separate contributions of sex and gender. Recent work has shown that sex and gender independently influence performance on cognitively sex-/gender-sensitive tasks (Cartier et al., 2024; Kheloui et al., 2023). While our decision aligns with most prior studies and allows for direct comparisons, a more inclusive approach would improve the interpretability of results. Although participants were asked to report both assigned sex and gender identity, they were categorized as cisgender based solely on matching responses. Future studies should explicitly ask participants whether they identify as cisgender to ensure greater accuracy.

Finally, a notable limitation was the generally low trust in the feedback provided, which may have attenuated the impact of the manipulation. The feedback only included information about relative performance (i.e., percentile rank), without offering item-level accuracy and explanations which are known to improve feedback effectiveness (Kluger & DeNisi, 1996; Nicol & Macfarlane-Dick, 2006; Shute, 2008; Wisniewski et al., 2020). Additionally, trust in feedback was measured only at the end of the study, rather than immediately following the feedback, potentially reducing the accuracy of the measure. Future research should examine how different feedback formats influence perceived credibility and the subsequent effects on self-perception and cognitive performance.

### Conclusions

In summary, the present study demonstrated that while feedback affected cognitive performance similarly across cisgender men and women, women were generally more susceptible to declines in self-beliefs, both in response to feedback and even in its absence. All forms of feedback significantly influenced self-beliefs, but only false positive and true negative feedback led to improvements in MRT performance. Importantly, the performance boost following false positive feedback was mediated by increased self-confidence, whereas the improvement following true negative feedback was unrelated to changes in self-perception. These findings suggest that the effects of feedback on cognitive performance operate through distinct mechanisms depending on both the accuracy and valence of the feedback.

False positive feedback may enhance performance via increased self-confidence and motivation, whereas true negative feedback may do so through a stress-related mechanism that temporarily boosts cognitive functioning, even as it undermines self-efficacy. More broadly, the results challenge the assumption that improving self-beliefs will necessarily lead to enhanced performance and highlight the complex, and sometimes decoupled, relationship between self-perception and cognitive performance.

Future research should investigate the conditions under which feedback can enhance both self-beliefs and performance, explore the roles of motivation and stress regulation, and adopt inclusive frameworks to better understand how sex and gender intersect with self-perception and cognitive ability.

## Data Availability

The full dataset is available on the Open Science Framework (https://osf.io/bd95q/; 10.17605/OSF.IO/BD95Q).

## Footnotes

1 Sex and gender are distinct concepts, yet growing evidence highlights the interplay of biological and social influences on cognitive performance. We use the term sex/gender because (1) in many previous studies, it is unclear whether significant findings reflect effects of sex, gender, or both, (2) prior work often does not specify whether assigned sex or gender identity served as the grouping variable, and (3) our sample consists of cisgender participants, for whom assigned sex and affirmed gender align, making the results equivalent whether analyzed by “sex” or “gender.” When referring to previous findings, we use the terms man and woman for clarity.
